# Safety and Efficacy of Landiolol Hydrochloride in Children with Tachyarrhythmia of Various Etiologies

**DOI:** 10.1007/s00246-021-02653-7

**Published:** 2021-06-07

**Authors:** Atsuko Ashida, Noriyasu Ozaki, Kanta Kishi, Yutaka Odanaka, Shintaro Nemoto, Hayato Konishi, Akira Ashida

**Affiliations:** 1 Department of Pediatrics, Osaka Medical and Pharmaceutical University, 2-7 Daigaku-machi, Takatsuki, Osaka 569-8686 Japan; 2 Department of Pediatric Thoracic and Cardiovascular Surgery, Osaka Medical and Pharmaceutical University, Takatsuki, Osaka Japan

**Keywords:** Landiolol hydrochloride, Tachyarrhythmia, Children, Rate control, Rhythm control, Congenital heart defects

## Abstract

The safety and efficacy of landiolol have not been fully elucidated in pediatric patients. This study aimed to clarify the safety and efficacy of landiolol in a pediatric cohort. We retrospectively assessed the clinical features of 21 pediatric patients who were administered landiolol at our hospital. We also investigated the rates of sinus rhythm conversion and heart rate response. The median patient age was 7 months (interquartile range 1–13 months). The etiology of tachyarrhythmia was junctional ectopic tachycardia in 10 patients (47.6%), atrial tachycardia in 10 patients (47.6%), and ventricular tachycardia in 1 patient (4.8%). Of the 21 children, 18 (85.7%) had congenital heart defects, including 14 (77.8%) in whom a landiolol infusion was performed perioperatively. The landiolol infusion was effective in 18 pediatric patients (85.7%), as measured by the conversion to sinus rhythm or a reduced heart rate. Atrial tachycardia in the perioperative period was terminated in all patients. Of 7 patients with tachyarrhythmias unrelated to the perioperative period, landiolol was effective in 5. No adverse effects were reported in any patient. Landiolol infusion is effective and safe in pediatric patients with tachyarrhythmia of various etiologies, especially those with atrial tachyarrhythmia during the perioperative period.

## Introduction

Landiolol is an ultra-short-acting beta-blocker that can be administered intravenously. In adult patients, the efficacy and safety of landiolol have been confirmed in large cohorts using a double blind-case-controlled study [1, 2]. Landiolol was initially indicated during the intra- and perioperative periods of cardiac surgery. However, the 2013 J-Land study reported that landiolol is also effective for perioperative tachyarrhythmia unrelated to cardiac surgery [2].

In contrast, few studies have described the use of landiolol in pediatric patients [3–7]. We believe that landiolol is effective and safe for pediatric patients and aimed to reveal the efficacy and safety through our single-center experience of administering landiolol to pediatric patients with tachyarrhythmia with various etiologies.

## Materials and Methods

We retrospectively reviewed the data of pediatric patients in whom an infusion of landiolol was performed to treat tachyarrhythmia between January 2009 and July 2020 at Osaka Medical College Hospital. We collected data regarding patient characteristics, congenital heart disease, the diagnosis and etiology of tachyarrhythmia, the type of cardiac surgery, the dose of the landiolol infusion, the pre-and post-infusion heart rate and blood pressure, and the conversion to sinus rhythm. The post-values were taken one hour after reaching the maximum dose of landiolol. We determined that landiolol was effective if the tachyarrhythmia was converted to sinus rhythm (achieved rhythm control) or the heart rate was reduced by more than 20% (achieved rate control) [4]. To evaluate the safety, we monitored and investigated the adverse events related to landiolol, such as bradycardia, arrhythmia, hypotension, cardiac dysfunction, hypoglycemia, and respiratory changes due to bronchial spasms.

We initiated the landiolol without a loading dose for all children of our cohort. The dose of initiation was 1 to 5 μg/kg/min. We titrated the dose up to 40 μg/kg/min if the efficacy was not sufficient and the adverse events were not detected. We did not change the protocol based on the etiology of the arrhythmia.

Continuous data are presented as mean ± standard deviation. Categorical data are presented as number (percentage). The pre-and post-infusion heart rate and blood pressure were compared using the paired *t* test. JMP Pro version 14 statistical software (SAS Institute Inc., Cary, NC, USA) was used for all statistical analyses. Statistical significance was set at *P* < 0.05.

The Institutional Review Board of the Osaka Medical College approved this study, and informed consent was obtained via opt-out due to the retrospective nature of this study.

## Results

Twenty-one pediatric patients with tachyarrhythmia of various etiologies were treated with landiolol from January 2007 to July 2020 at Osaka Medical College Hospital. Patient characteristics are shown in Table 1. The median age at the initiation of landiolol was 7 months (interquartile range (IQR) 1–13 months). The median body weight was 6.8 kg (IQR 4–8.6 kg). Eighteen patients (85.7%) had congenital heart disease, and 14 (66.7%) patients had perioperative tachyarrhythmia related to cardiac surgery. Ten patients (47.6%) were diagnosed with atrial tachycardia (AT), 10 patients (47.6%) with junctional ectopic tachycardia (JET), and 1 patient (4.8%) with ventricular tachycardia. Perioperative tachyarrhythmia related to cardiac surgery occurred in 6 patients (60%) with AT and 8 patients (80%) with JET.

**Table 1:** Patient demographics

Patient	Age (months)	Sex	Body weight (kg)	Diagnosis of congenital heart defects	Related to cardiac surgery	Etiology of arrhythmia
1	91	M	15	SLV, PA s/p TCPC	No	AT
2	7	M	8	PAIVS, s/p BTS s/p RVOTR	Yes	AT
3	50	M	18	Normal heart	No	AT
4	1	F	3.6	HLHS s/p Norwood operation	Yes	AT
5	3	F	4.7	TAPVC (supracardiac type) s/p TAPVC repair	Yes	AT
6	13	M	7.2	Noonan syndrome, ASD secundum type, vPS s/p ASD closure, pulmonary valvotomy	Yes	AT
7	0.8	M	3.3	TGA with VSD s/p arterial switch operation	Yes	AT
8	1	M	4.4	Normal heart	No	AT
9	9	F	8.5	Rupture of the mitral chordae tendineae with massive mitral valve regurgitation	Yes	AT
10	33	F	10.2	Trisomy 18, VSD s/p PAB, s/p ICR	No	AT
11	26	F	11	RIH, SV, PA, TAPVC s/p TAPVC repair	No	JET
12	0.4	M	2.5	RIH, SA, SV, TAPVC s/p TAPVC repair	Yes	JET
13	2	F	4	SRV, DORV, ASD s/p Norwood type operation	Yes	JET
14	12	F	5.1	RIH, SRV, s/p BDG operation	No	JET
15	4	M	5.1	Trisomy 21, cAVSD	Yes	JET
16	22	F	8.6	Trisomy18, VSD s/p PAB, s/p ICR	Yes	JET
17	6	M	8.1	TOF s/p total correction	Yes	JET
18	8	M	6.8	TOF s/p total correction	Yes	JET
19	7	M	8.8	Dextrocardia, DORV, vPS, VSD s/p ICR	Yes	JET
20	0.3	F	3	TGA with IVS	Yes	JET
21	0.6	F	3.5	Normal heart	No	VT

*M* male, *F* female, *SLV* single left ventricle, *PA* pulmonary atresia, *TCPC* total cava pulmonary connection, *PAIVS* pulmonary atresia with intact ventricular septum, *BTS* Blalock-Taussig shunt, *RVOTR* right ventricular outflow reconstruction, *HLHS* hypoplastic left heart syndrome, *TAPVC* total anomalous of pulmonary vein connection, *ASD* atrial septal defect, *vPS* valvular pulmonary stenosis, *TGA* transposition of great arteries, *VSD* ventricular septal defect, *PAB* pulmonary artery banding, *ICR* intracardiac repair, *RIH* right isomerism heart, *SV* single ventricle, *SA* single atrium, *SRV* single right ventricle, *DORV* double-outlet right ventricle, *BDG* bidirectional Glenn, cAVSD complete atrioventricular defect, *TOF* tetralogy of Fallot, *IVS* intact ventricular septum, *AT* atrial tachycardia, *JET* junctional ectopic tachycardia, *VT* ventricular tachycardia

Table 2 shows the dose of landiolol and the response to landiolol in each patient. The median maximum dose of landiolol was 11 μg/kg/min (IQR 8–19.5 μg/kg/min). Sinus conversion (rhythm control) was achieved in 14 patients (66.7%), and heart rate reduction (rate control) was achieved in four patients (19.0%) in whom sinus conversion could not be achieved; therefore, landiolol was effective in 18 patients (85.7%). Landiolol was effective in 71.4% of patients with tachyarrhythmia not related to cardiac surgery. Landiolol successfully controlled AT in all patients and JET in 8 patients (80%). Landiolol was effective in 50% of patients with JET unrelated to cardiac surgery and in 87.5% of patients with JET related to cardiac surgery. Landilol could not terminate VT in our cohort. There were no adverse events related to landiolol infusion in any patient.

**Table 2:** Dose and effectiveness of landiolol infusion

Patient	Maximum dose of landiolol hydrochloride (μg/kg/min)	Sinus conversion	Achieved rate control	Effectiveness of landiolol	Pre-infusion heart rate (beats per minute)	Post-infusion heart rate (beats per minute)	Pre-infusion blood pressure (mmHg)	Post-infusion blood pressure (mmHg)
1	4	Yes	Yes	Yes	130	80	86	90
2	5	Yes	Yes	Yes	200	150	96	94
3	40	No	Yes*	Yes	220	160	100	102
4	12.5	Yes	Yes	Yes	160	120	60	66
5	3.7	Yes	Yes	Yes	200	140	80	76
6	11	Yes	Yes	Yes	230	130	68	86
7	5.6	Yes	Yes	Yes	190	150	70	78
8	5	Yes	Yes	Yes	210	130	85	90
9	21	Yes	Yes	Yes	220	130	80	90
10	40	No	Yes*	Yes	200	160	95	95
11	10	Yes	Yes	Yes	200	90	88	102
12	10	No	Yes*	Yes	200	160	65	70
13	8	Yes	Yes	Yes	210	150	70	70
14	30	No	No	No	230	230	75	70
15	15	Yes	Yes	Yes	180	120	67	80
16	9.5	Yes	Yes	Yes	170	120	77	119
17	40	No	No	No	230	230	69	67
18	15	Yes	Yes	Yes	180	130	81	111
19	19.5	No	Yes*	Yes	185	140	76	67
20	10	Yes	Yes	Yes	207	145	87	75
21	19	No	No	No	130	130	88	90

No adverse events were reported in any patient

Rate control is defined as a ≥ 20% reduction in heart rate

*Sinus conversion was not achieved, but rhythm control could be achieved by landiolol infusion

**Fig. 1:**
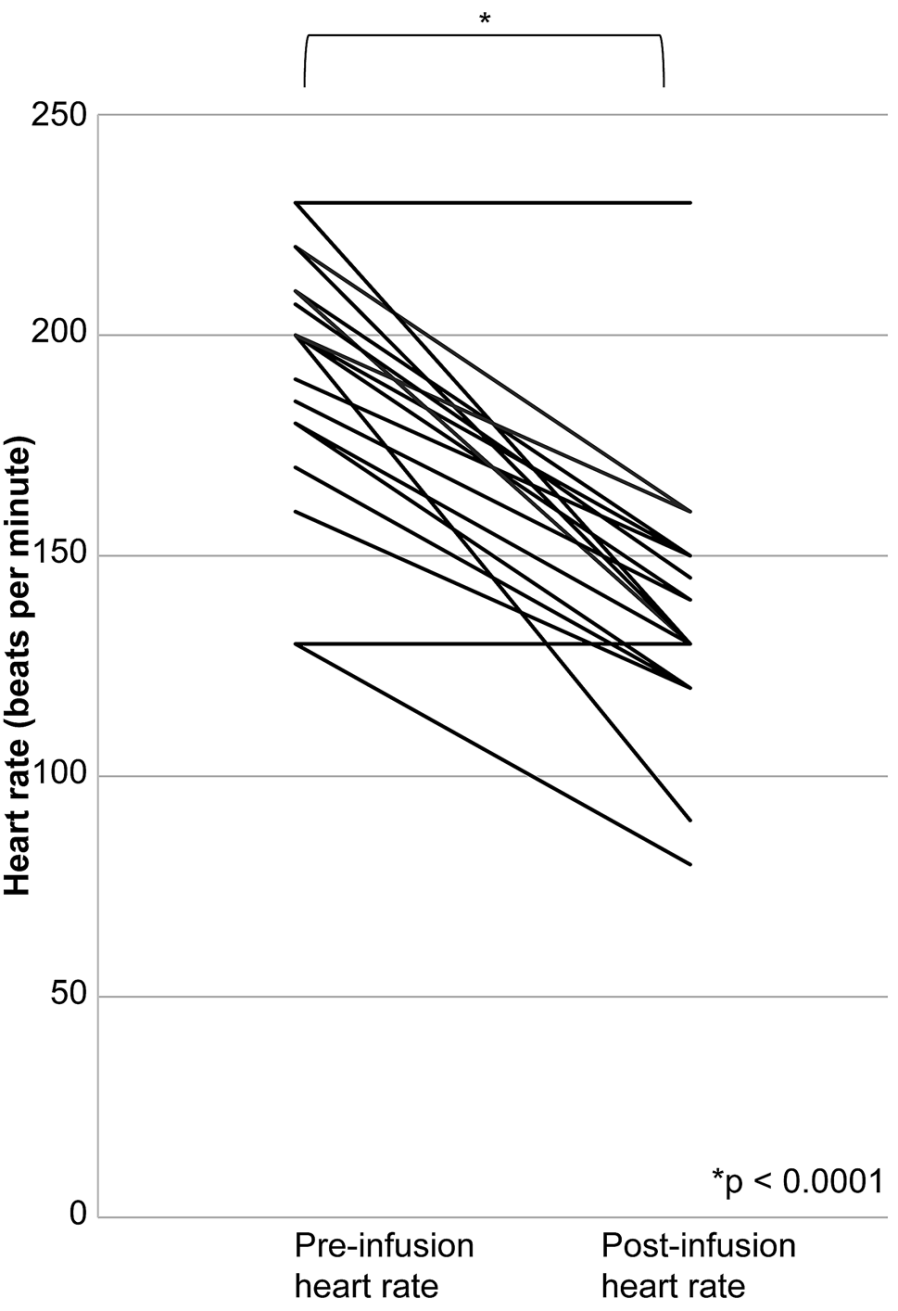
Heart rate, before and after landiolol infusion. The changes in heart rate before and after the infusion of landiolol hydrochloride are shown. Landiolol significantly reduced the heart rate

**Fig. 2:**
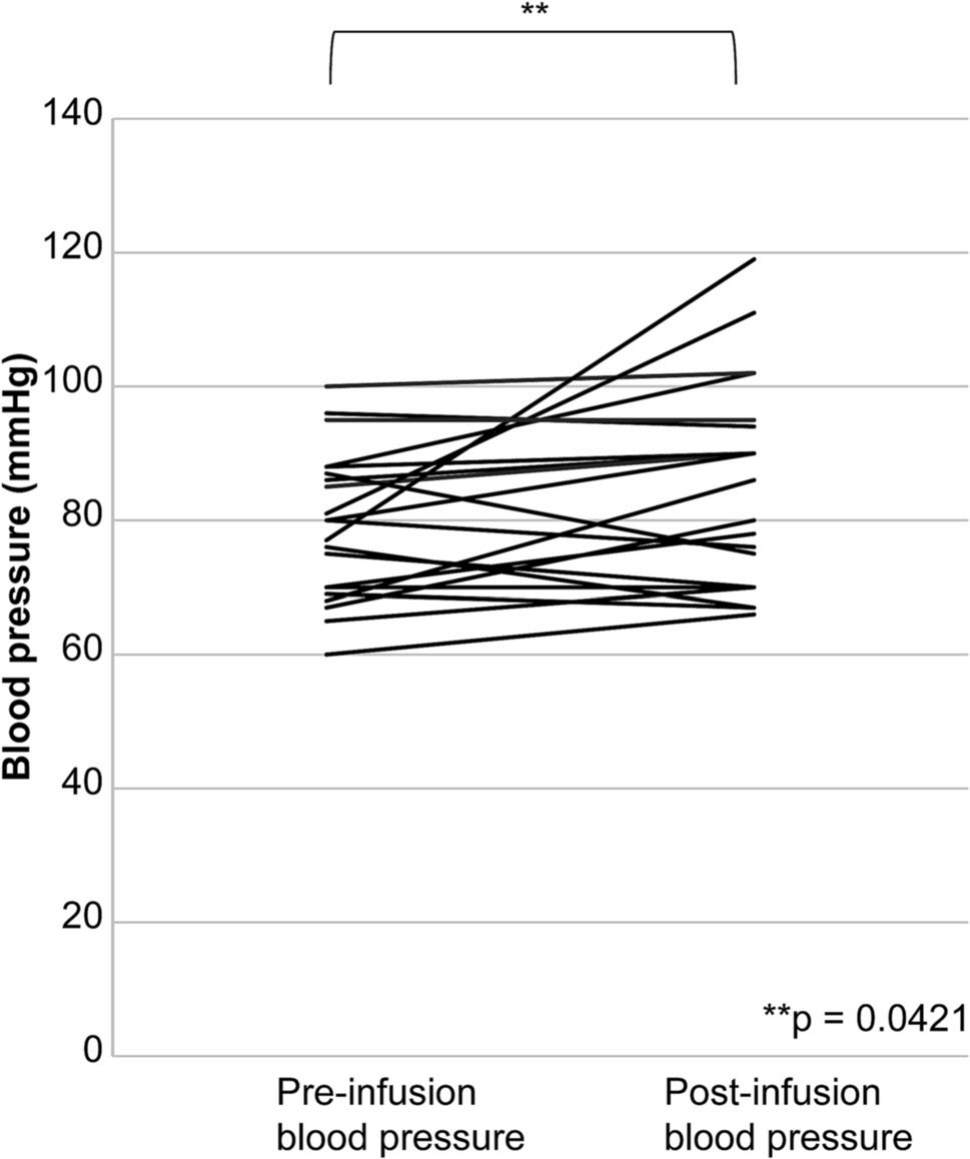
Blood pressure before and after landiolol infusion. The blood pressures before and after the infusion of landiolol hydrochloride are shown. Landiolol significantly increased the blood pressure

The infusion of landiolol significantly reduced the heart rate (*p* < 0.0001; Fig. 1) and significantly increased the blood pressure (*p* = 0.0421; Fig. 2).

## Discussion

Landiolol effectively controlled the heart rhythm or rate in 18/21 pediatric patients (85.7%) in this study with no adverse events. All patients with AT were effectively treated, and landiolol was also effective in patients with JET, especially in the postoperative period.

### Efficacy of Landiolol in the Perioperative and Non-perioperative Periods

Few studies have described the efficacy of landiolol in pediatric patients [3–7], and those studies reported patients with tachyarrhythmia in the perioperative period of cardiac surgery. The efficacy of landiolol in pediatric patients in settings other than the postoperative period has only been reported in case reports [8, 9]. This study reports the efficacy of landiolol in pediatric patients in the postoperative period and at times unrelated to cardiac surgery, as it includes seven patients who did not undergo surgery and received landiolol. Landiolol was found to be effective in five patients (71.4%) who did not undergo surgery and in 13 patients (92.9%) who underwent cardiac surgery. These results suggest that landiolol is more effective for tachyarrhythmia related to cardiac surgery in pediatric patients, especially during the perioperative period. The increase of intrinsic catecholamines can induce tachyarrhythmia, including AT and JET postoperatively, due to an increased automaticity mechanism. Landiolol can reduce the efficacy of intrinsic catecholamines that induce JET or AT [10]. However, tachyarrhythmia with an arrhythmogenic substrate may be refractory to landiolol. We could not achieve rhythm control in some children of our cohort. The etiology of arrhythmia in those children might be related to the substrate, such as re-entry mechanisms. Landiolol suppresses the conduction of the atrioventricular node and reduces the ventricular rate that can lead to achieved rate control for arrhythmias with the reentrant mechanism. However, landiolol cannot suppress reentrant arrhythmias and not achieve rhythm control for those because landiolol suppresses the onset of action potentials but does not prolong the refractory period.

### Comparing Landiolol and Esmolol

Landiolol is an ultra-short-acting and beta-one selective beta-blocker that is administered intravenously [11, 12]. Landiolol is produced by a Japanese company and is available in Japan. The half-life of landiolol is approximately four minutes, which is the shortest among beta-blocker compounds to date. Landiolol has a strong affinity to the beta-one receptor, which is the most dominant beta-adrenergic receptor in the heart. Landiolol has high selectivity to beta-one adrenergic receptors with a ß1/ß2 ratio of 255. Esmolol is another ultra-short-acting and cardio-selective beta-adrenoreceptor blocker that is available worldwide [13, 14]. Compared with esmolol, landiolol has a shorter half-life and better affinity to beta-one adrenoreceptors [15]. While esmolol can result in hypotension, landiolol does not [16]; however, some studies have reported that esmolol is more effective at lowering blood pressure than landiolol [17, 18]. The serum level of landiolol must be high enough to terminate tachyarrhythmia [11, 12]. The short half-life of landiolol allows for a rapid infusion of a sufficient dose to achieve sufficient serum levels. The potent cardio-selectivity of landiolol decreases the risk of hypotension, even at high doses. Therefore, landiolol is applicable in pediatric patients with low cardiac function postoperatively or tachycardia-induced cardiomyopathy.

From the standpoint of the anti-arrhythmic effect, esmolol might be somewhat weak. Trippel et al. reported that the effect of esmolol to suppress sustained supraventricular tachycardia was recognized in only one out of 4 children with reentrant supraventricular tachycardia. Regarding automatic supraventricular tachycardia, esmolol could not suppress tachycardia in a child with an automatic mechanism [19]. Therefore, we consider that landiolol is more efficient and safe than esmolol for children, especially in the perioperative setting.

### Landiolol in Adult Patients Compared to Pediatric Patients

The J-Land study investigated the efficacy of landiolol in adult patients with atrial fibrillation and impaired left ventricular function and found that landiolol effectively controls the heart rate in these patients [2]. According to previous studies, the rate of sinus rhythm conversion (achieved rhythm control) is 2.2–26% [1, 2]. In this study, the rate of sinus rhythm conversion was 66.7%. This discrepancy between children and adults regarding the rate of rhythm control could be originated from the difference in the etiologies of arrhythmias. The etiologies were junctional ectopic tachycardia and atrial ectopic tachycardia for children of our cohort and atrial fibrillation and atrial flutter for adult cases. Depending on the patient’s clinical status and arrhythmic etiology, rate control may be the target treatment for tachyarrhythmia in adult patients. However, in pediatric patients with fragile hemodynamics (such as after cardiac surgery), rhythm control improves the fragile hemodynamics, suggesting that landiolol would be effective as a first-line treatment in these patients.

### Safety-Related Features of Landiolol

Due to landiolol’s ultra-short half-life, adverse effects can be avoided by stopping the infusion [11, 12]. Other anti-arrhythmic agents, such as amiodarone, flecainide, and sotalol have also been reported as effective in pediatric patients [20–25]. However, these drugs have longer half-lives and, therefore, adverse effects cannot be stopped as quickly as the adverse effects of landiolol. Moreover, landiolol exhibits beta-one adrenoreceptor selectivity, which reduces the adrenergic effect of intrinsic catecholamines on the heart rate without the negative inotropic effects on cardiac and other tissues.

### Limitations

This retrospective study was conducted using data from a single center, and therefore, included a small number of patients. The actual effect of landiolol could not be evaluated in this study due to the retrospective design. While we did not restrict the criteria for landiolol infusion, the possibility of a selection bias cannot be ruled out.

## Conclusion

Landiolol is effective and safe in pediatric patients with tachyarrhythmia of various etiologies, including those unrelated to cardiac surgery. Landiolol is most effective during the perioperative period in pediatric patients with AT.

## Data Availability

The database generated and analyzed during the current study are proprietary and cannot be shared under the conditions of our data use agreement.

## Code Availability

Microsoft Office, JMP Pro version 14 statistical software (SAS Institute Inc., Cary, NC, USA).
